# Investigating Splice Defects in *USH2A* Using Targeted Long-Read Sequencing

**DOI:** 10.3390/cells13151261

**Published:** 2024-07-26

**Authors:** Shwetha Chandrasekhar, Siying Lin, Neringa Jurkute, Kathryn Oprych, Leire Estramiana Elorrieta, Elena Schiff, Samantha Malka, Genevieve Wright, Michel Michaelides, Omar A. Mahroo, Andrew R. Webster, Gavin Arno

**Affiliations:** 1 UCL Institute of Ophthalmology, University College London, London EC1V 9EL, UK; 2 National Institute of Health Research Biomedical Research Centre at Moorfields Eye Hospital and the UCL Institute of Ophthalmology, London EC1V 9EL, UK; 3 Department of Neuro-Ophthalmology, The National Hospital for Neurology and Neurosurgery, London WC1N 3BG, UK; 4 Clinical Genetics, St George’s University Hospitals NHS Foundation Trust, London SW17 0QT, UK; 5 Section for Paediatrics, Department of Infectious Diseases, Faculty of Medicine, Imperial College London, London W2 1NY, UK; 6 Department of Ophthalmology, St Thomas’ Hospital, London SE1 7EH, UK; 7 Greenwood Genetic Center, Greenwood, SC 29646, USA

**Keywords:** *USH2A*, inherited retinal disease, long-read sequencing, Oxford nanopore sequencing, nasal epithelial cells, deep intronic variant, cryptic splice-altering variant

## Abstract

Biallelic variants in *USH2A* are associated with retinitis pigmentosa (RP) and Type 2 Usher Syndrome (USH2), leading to impaired vision and, additionally, hearing loss in the latter. Although the introduction of next-generation sequencing into clinical diagnostics has led to a significant uplift in molecular diagnostic rates, many patients remain molecularly unsolved. It is thought that non-coding variants or variants of uncertain significance contribute significantly to this diagnostic gap. This study aims to demonstrate the clinical utility of the reverse transcription–polymerase chain reaction (RT-PCR)–Oxford Nanopore Technology (ONT) sequencing of *USH2A* mRNA transcripts from nasal epithelial cells to determine the splice-altering effect of candidate variants. Five affected individuals with USH2 or non-syndromic RP who had undergone whole genome sequencing were recruited for further investigation. All individuals had uncertain genotypes in *USH2A*, including deep intronic rare variants, c.8682-654C>G, c.9055+389G>A, and c.9959-2971C>T; a synonymous variant of uncertain significance, c.2139C>T; p.(Gly713=); and a predicted loss of function duplication spanning an intron/exon boundary, c.3812-3_3837dup p.(Met1280Ter). In silico assessment using SpliceAI provided splice-altering predictions for all candidate variants which were investigated using ONT sequencing. All predictions were found to be accurate; however, in the case of c.3812-3_3837dup, the outcome was a complex cryptic splicing pattern with predominant in-frame exon 18 skipping and a low level of exon 18 inclusion leading to the predicted stop gain. This study detected and functionally characterised simple and complex mis-splicing patterns in *USH2A* arising from previously unknown deep intronic variants and previously reported variants of uncertain significance, confirming the pathogenicity of the variants.

## 1. Introduction

Biallelic variants in *USH2A* are associated with both Usher Syndrome Type 2 (USH2), characterised by hearing loss followed by retinitis pigmentosa (RP), as well as non-syndromic RP (nsRP). Both conditions follow an autosomal recessive mode of inheritance, with progressive degeneration of the rod followed by cone photoreceptors leading to progressive nyctalopia (night blindness) and visual field constriction, which can progress to central vision loss in more severe cases. Where USH2A function is severely impaired, [for instance, with biallelic loss of function (LOF) variants], moderate-to-severe early-onset hearing impairment is also present, leading to USH2 (first described by Albrecht von Gräfe in 1858) [1,2,3,4].

Variants in *USH2A* are the most common cause of autosomal recessive RP (arRP) in the Moorfields Eye Hospital inherited retinal disease patient cohort and the second most common cause of inherited retinal disease overall, accounting for over 8% of all affected families [5,6]. It is estimated that a third of disease-causing variants impact pre-mRNA splicing [7]. Whole genome sequencing (WGS) has the capability to detect these variants; however, unless they affect a canonical splice site, or have previously been established as pathogenic or likely pathogenic according to the Association for Clinical Genomic Science UK (ACGS) [8] guidelines, their clinical significance can be challenging to ascertain. Consequently, these cryptic splice-altering variants are often overlooked by clinical diagnostic analysis pipelines and likely contribute to a number of affected individuals remaining without a complete molecular diagnosis [9,10]. These patients are subsequently unable to access personalised clinical care, including accurate prognoses and genetic counselling, family planning options, and eligibility for emerging gene therapies [11,12]. For many affected individuals, a definite molecular diagnosis also contributes significantly to their emotional well-being by providing clarity and a sense of empowerment regarding their condition [13].

This study applies a novel approach to investigate candidate splice-altering variants in the *USH2A* gene identified via WGS. We assessed five candidate variants, all predicted using in silico algorithms to affect splicing, and validated the effect of these variants on pre-mRNA splicing through RT-PCR-ONT transcript sequencing, providing functional evidence to support pathogenicity. We further highlight the practical application of this approach, with a rapid turnaround time supporting its clinical utility and potential for integration into clinical laboratory diagnostic pipelines.

## 2. Materials and Methods

### 2.1. Patient Cohort

A cohort of over 2300 patients with inherited retinal disease seen within the Moorfields Eye Hospital Inherited Eye Disease Service (1600 through the UK 100,000 Genomes Project; 100 kGP, and 722 through the NIHR Bioresource rare disease project) were investigated using a robust clinical pipeline, comprising Illumina WGS and a subsequent bioinformatic analysis focused on protein-altering variants within an applied gene panel tailored to the patient’s clinical phenotype [10,14,15]. For patients included in this study, the analysis included variants in genes in the PanelApp R32 Retinal Disorders panel (v3.0, 247 genes) and the R67 Monogenic Hearing Loss panel (v3.0, 135 genes) where appropriate [15]. These variants were then classified according to the ACGS guidelines for variant interpretation [8], and only variants classified as pathogenic or likely pathogenic were considered by the clinical pipeline.

Patients that remained unresolved following this clinical pipeline underwent further evaluation at multi-disciplinary team meetings, which included inherited eye disease specialists, clinical and research scientists, and genetic counsellors. A comprehensive review of the patient’s clinical history, phenotype, and genomic data was performed to target further variant investigations [9]. For this study, patients with a clinical diagnosis of USH2 were selected, and variants in the three genes known to cause USH2 (*USH2A*, *ADGRV1*, and *WHRN*) received further scrutiny. Additionally, we also included patients with nsRP, where a pedigree analysis was consistent with an autosomal recessive mode of inheritance, and where the clinical analysis pipeline had identified a single pathogenic or likely pathogenic variant in *USH2A*, for further analysis.

### 2.2. Variant Analysis

#### 2.2.1. Variant Filtering

The variant analysis focused on rare variants [minor allele frequency of <0.001 in the gnomAD population datasets (v.4.1.0) and the Genomics England rare disease cohort] within the *USH2A*, *ADGRV1*, or *WHRN* genes, with additional filtering for call quality and zygosity consistent with the expected autosomal recessive mode of inheritance. For any candidate variants of interest identified, a comprehensive literature review was performed to identify any previous reports, and a search of the Genomics England and the NIHR BioResource rare disease cohorts was undertaken to identify any additional carriers with an nsRP or USH2 phenotype.

#### 2.2.2. In Silico Predictions

SpliceAI was used to predict the effect of variants on splicing [16]. SpliceAI computes a prediction (Δ) score ranging from 0 to 1, based on the probability of the variant affecting splicing. Variants with a score of 0.2 or more (classified as high-recall) were chosen for functional investigation. Candidate variants with a strong acceptor and donor gain prediction were further analysed for potential pseudoexon inclusion using NetGene2 [17] with a partner splice site of >0.60 within 500 bp considered as a possible pseudoexon signal.

### 2.3. Cell Collection, RNA Extraction, and RT-PCR

Nasal epithelial cells (NECs) were collected by gently swabbing the inferior turbinate epithelium using a cytology brush, with RNA extraction carried out following established protocols (Qiagen RNeasy Mini Kit Protocol: Purification of Total RNA from Animal Cells Using Spin Technology) [18].

RT-PCR was performed using random hexamers at a final concentration of 2.5 ng/µL (RT) followed by oligonucleotide primer pairs designed to span the region of interest in the *USH2A* mRNA transcript (NM_206933.4), covering the surrounding 4–7 exons, to ensure the detection of an array of possible splice alteration(s) caused by the candidate *USH2A* variant under investigation.

### 2.4. ONT Sequencing

Library preparation was carried out according to established ONT protocols, including end preparation, native barcoding (for samples multiplexed on a single run), and adapter ligation. Sequencing was performed on the Flongle Flow Cell using the MinKnow workflow and software (v22.05.5), with the base-calling and alignment of sequencing data performed using MinKNOW, Guppy v5.0.16, Minimap2 v2.22, and Samtools v1.9 bioinformatics toolkits and workflows. The resulting .BAM output files were visualised on Integrated Genome Viewer (IGV v2.14.1); splice alterations were visually inspected on reads, and sashimi plots were generated to show junctional fragments across splice junctions.

## 3. Results

Detailed patient data is presented for all 5 patients in the study, along with results of *USH2A* variant analysis. Table 1 outlines the *USH2A* variants selected for investigation, Table 2 details the *USH2A* genotypes of all the patients in the study, and Table 3 presents SpliceAI predictions for the *USH2A* variants under investigation.

### 3.1. Patient 1 (GC20114)

Patient 1 is a 33-year-old White Caucasian male with a clinical diagnosis of USH2. He had a history of hearing loss in childhood and has been using hearing aids since the age of 1. In his early 20s, he began experiencing night and peripheral vision difficulties. A retinal examination identified changes consistent with RP, leading to a diagnosis of USH2. There is no known family history of hearing loss or eye disease.

WGS and a subsequent clinical variant analysis revealed a single heterozygous pathogenic variant in *USH2A*: c.6289_6302del14; p.(Ile2097Ter). A non-coding variant analysis identified an additional deep intronic *USH2A* variant of interest—c.9959-2971C>T—with parental segregation confirming that both variants were inherited in trans. This variant, located within intron 50, was absent from gnomAD data, not previously reported in any patients, and displayed high SpliceAI (Δ) scores for donor (GCATG>GTATG) and upstream acceptor activation (Table 3) suggestive of possible pseudoexon insertion; it was selected for further functional investigation.

**Figure 1:**
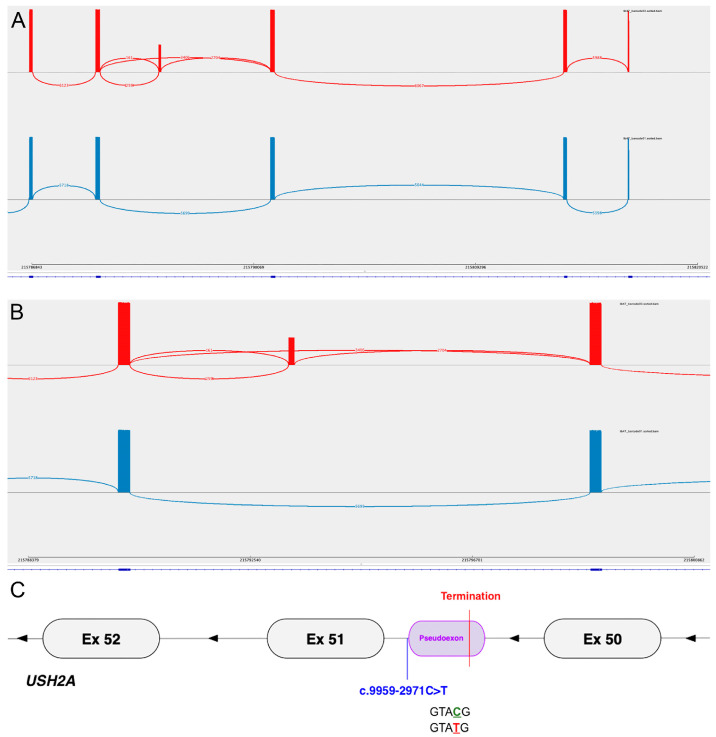
(**A**) Sashimi plot from IGV showing the splicing junctions of Patient 1 (top, red) and a control (bottom, blue). This fragment includes exons 48–52 of *USH2A* from right to left (reverse strand, 3′ to 5′). Each bar represents an exon, and the height of the bars represents the read depth. The control (bottom, blue) shows the expected canonical splicing. Patient 1 (top, red) shows mis-splicing, resulting in the retention of a pseudoexon comprising 113 bp derived from intron 50. The reference *USH2A* transcript (NM_206933.4) is represented at the bottom of the track. (**B**) Enlarged view of sashimi plot showing exons 50–51 from (**A**). (**C**) Graphical representation of the mis-splicing in Patient 1 relative to the variant location and showing the mutant donor site motif.

The RT-PCR and ONT sequencing of an 863 bp transcript region spanning *USH2A* exons 48–52 revealed a pseudoexon insertion of 113 bp following exon 50, predicted to lead to a premature termination 8 codons after the start of the pseudoexon (Figure 1). This sequence was consistent with the predicted consequence and was seen in 44.3% of reads (2704/6110) having a junction at the end of exon 50, in keeping with the heterozygosity of c.9959-2971C>T. Notably, 5.9% (161/2704) of these reads show the additional mis-splicing of exon 51, due to the apparent activation of a possible alternate acceptor site 20 bp downstream of the canonical exon 51 splice acceptor motif. The observed frameshift consequence of this variant in trans with a LOF variant is in keeping with the USH2 clinical diagnosis reported for this patient.

### 3.2. Patient 2 (GC19437)

Patient 2 is an 80-year-old White Caucasian female, clinically diagnosed with USH2. She has two sisters who are similarly affected and another sister is unaffected. WGS and a subsequent clinical pipeline analysis identified heterozygosity for a known pathogenic deep intronic variant in *USH2A* (c.7595-2144A>G, ClinVar ID: 30722), along with a rare synonymous *USH2A* variant, c.2139C>T; p.(Gly713=). Both variants were also identified in a heterozygous form in her two affected sisters. The p.(Gly713=) variant had previously been classified as a VUS in ClinVar [although it has recently been re-classified as pathogenic by Invitae in December 2023 following a recent publication describing this variant in combination with a *USH2A* missense variant c.1859G>T; p.(Cys620Phe) in a patient with USH2 [19]. It was also detected in another family with nsRP within the 100 kGP rare disease cohort, in trans, with a second pathogenic *USH2A* variant. In light of these observations, and the high SpliceAI (Δ) score associated with this variant, it was prioritised for functional investigation.

**Figure 2:**
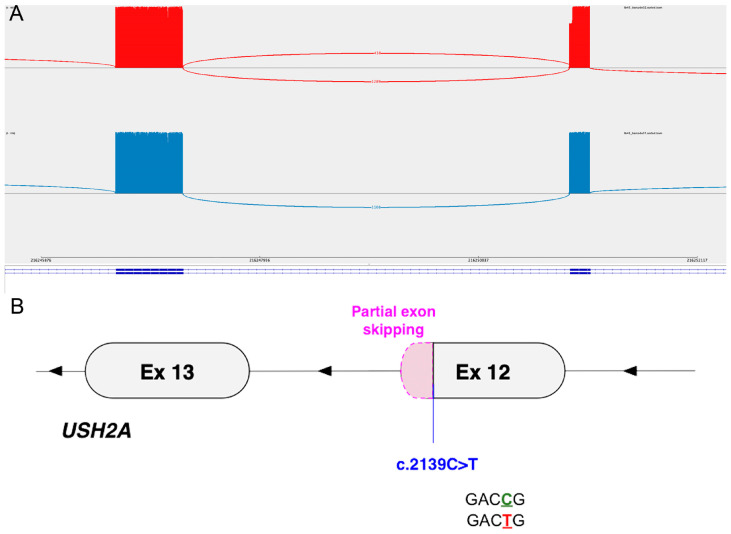
(**A**) Sashimi plot from IGV showing splice junctions for *USH2A* exons 12 and 13 of Patient 2 (top, red) and a control (bottom, blue). Exons are depicted from right to left (reverse strand, 3′ to 5′) with each bar depicting an exon and the height of the bars indicating the number of reads. The control (bottom, blue) exhibits the expected normal splicing. In contrast, Patient 2 (top, red) shows mis-splicing (partial exon 12 skipping) due to the variant. The reference *USH2A* transcript (NM_206933.4) is represented by the blue line at the bottom. (**B**) Graphical representation of the mis-splicing pattern in Patient 2 relative to the variant location and the mutant sequence.

The RT-PCR and ONT sequencing of a 1001 bp transcript region of *USH2A* spanning exons 10–15 in Patient 2 showed mis-splicing—the creation of a novel donor site (GCCAG>GTCAG) leading to partial exon 12 skipping was visible in 26.7% of the reads (492/1840) and would lead to an in-frame deletion of 30 bases. Additionally, a small proportion of reads (2.6%, 35/1348) was found to have read mapping junctions at another alternate donor site in exon 12, predicted to lead to a frameshift and premature termination [p.(Cys715AlaTer2)]. These mis-splicing events were not identified in a control sample (Figure 2).

The fact that only 26.7% of reads showed the splice defect may suggest that either the effect on splicing only affects approximately half of the transcripts arising from the mutant allele, the mutant transcript may be subjected to nonsense-mediated decay (NMD), or there is preferential amplification for the wild-type allele in the PCR. To test the first, an analysis of the base-call at position 2139 showed that of the normally spliced transcripts, only 1.4% (20/1413) had the variant call, suggesting that the normal splicing from the mutant allele was not contributing to the reduced proportion. The effect of the partial loss of exon 12 on the USH2A protein is unclear, although it may be expected to be significantly damaging given the patient’s clinical diagnosis of USH2.

### 3.3. Patient 3 (GC2569)

Patient 3 is a 79-year-old Indian female with nsRP; she also has an affected son with nsRP. WGS identified two predicted LOF variants in *USH2A* inherited in trans: c.4222C>T; p.(Gln1408Ter) and c.3812-3_3837dup; p.(Met1280Ter); her affected son was found to be a heterozygous carrier for the c.3812-3_3837dup variant only (he was later found to have a homozygous pathogenic variant in EYS accounting for his arRP). Biallelic LOF variants in *USH2A* typically cause USH2 with congenital or early-childhood-onset hearing loss. Given that Patient 3 retained normal hearing into her 70s, it was hypothesised that one of the two *USH2A* variants identified might not lead to complete LOF; the more likely candidate was thought to be the c.3812-3_3837dup variant, which spans a splice junction and has conflicting classifications for pathogenicity on ClinVar (ID: 420502). Consequently, this variant was selected for functional analysis.

**Figure 3:**
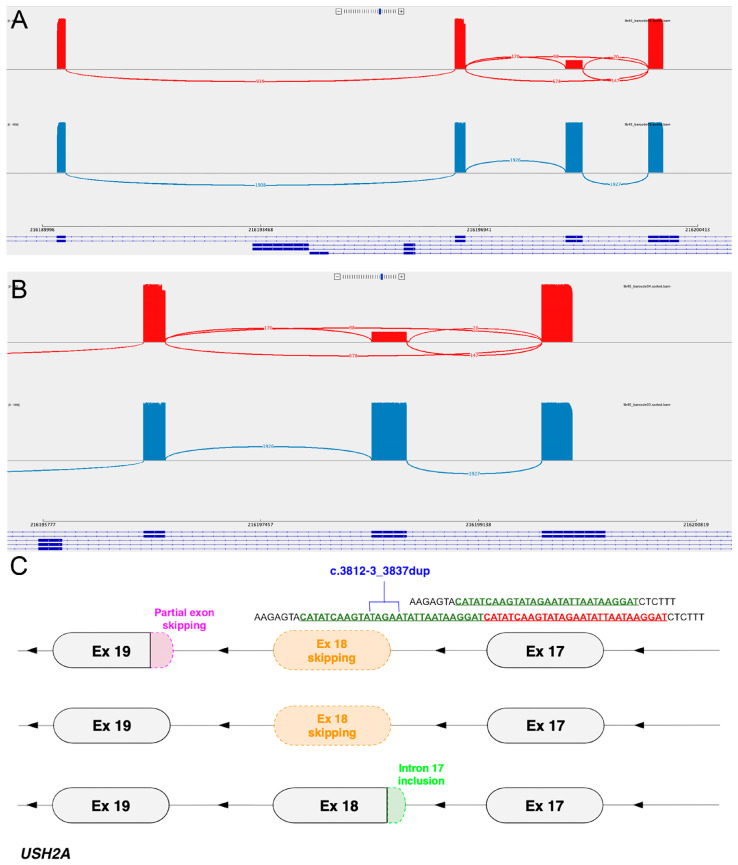
(**A**) Sashimi plot showing the splice junctions of Patient 3 (red, top) and a control (bottom, blue). *USH2A* exons 17–20 are depicted from right to left (reverse strand, 3′ to 5′). The patient sample (top, red) shows multiple mis-splicing events—the inclusion of the duplicated nucleotides (mapping as partial intron 17 inclusion) and exon 18 skipping with or without partial exon 19 skipping. The control sample (bottom, blue) shows canonical splicing. (**B**) Enlarged view of exons 17–19 from (**A**). (**C**) Graphical representation of the multiple mis-splicing events identified in Patient 3.

RT-PCR and ONT sequencing was performed on an 828 bp transcript region spanning exons 17-20 of *USH2A*, which revealed the skipping of exon 18 in 60.9% of reads (678/1113), the skipping of exon 18 and partial exon 19 in 8.8% of reads (98/1113), and a low proportion of reads (20/1113, 1.8%) showing the abnormal splicing use of the most upstream splice junction (Figure 3); these mis-splicing events were not identified in a control sample (Figure 3). Both exon-skipping mis-splicing events (exon 18 skipping and exon 18/partial exon 19 skipping) are in-frame, whilst the partial intron 17 inclusion detected at low levels would lead to the predicted p.(Met1280Ter).

### 3.4. Patient 4 (GC20993)

Patient 4 is a 60-year-old White Caucasian female with childhood-onset nsRP; there was no known family history of hearing loss or eye disease. WGS and the subsequent clinical analysis pipeline analysis identified a heterozygous pathogenic variant in *USH2A*—c.9882C>G; p.(Cys3294Trp), (ClinVar ID: 438036). Following a variant analysis, a single candidate variant with a SpliceAI (Δ) score was identified—c.8682-654C>G. This variant showed a strong donor site gain (GTCAG>GTGAG) prediction with upstream acceptor activation similar to that observed for Patient 1, suggesting possible 130bp pseudoexon insertion, and was consequently chosen as the candidate variant.

**Figure 4:**
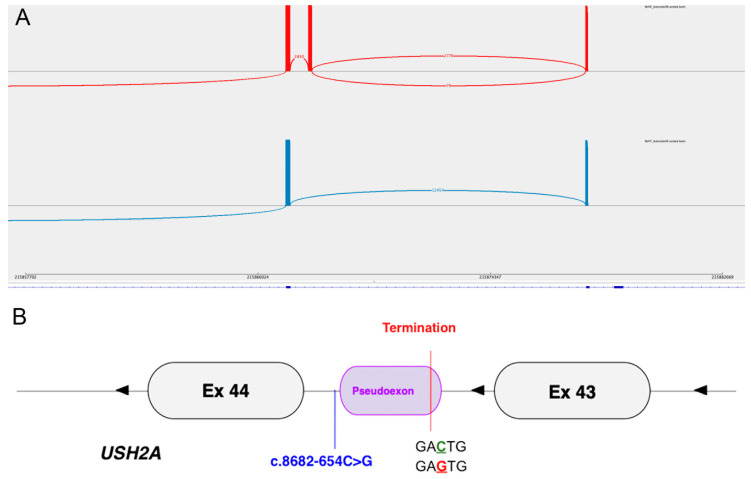
(**A**) Sashimi plot showing the splice junctions of Patient 4 (red, top) and a control (bottom, blue). *USH2A* exons 43–44 are depicted from right to left (reverse strand, 3′ to 5′), with each bar representing an exon and the height of the bars indicating the number of reads. The control (bottom, blue) shows canonical splicing only. The patient (top, red) shows mis-splicing—a pseudoexon inclusion of 130 bp. The reference *USH2A* transcript (NM_206933.4) is represented by the blue line at the bottom. (**B**) Graphical representation of the mis-splicing patterns in Patient 4, showing the mutant donor site motif.

RT-PCR and ONT sequencing was performed across a 702 bp transcript region encompassing *USH2A* exons 43–47 to assess the impact of c.8682-654C>G on splicing. The read data showed a pseudoexon insertion matching the predicted sequence and predicted the result of a premature termination at the second codon of the pseudoexon (Figure 4). Despite heterozygosity for the candidate intronic variant, the pseudoexon insertion was observed in 100% of the sequencing reads (2830/2830); given the trans variant is a missense and is not predicted to cause the NMD of that allele (with a possible resulting low amplification), we hypothesised that this observation could be due to preferential allele amplification.

Given the clinical phenotype and *USH2A* genotype observed in Patient 4, it is difficult to assess the severity of the deep intronic variant. The predicted effect is LOF due to the stopgain at the first altered codon (p.Phe2895Ter). The second allele, p.(Cys3294Trp), is enriched in nsRP cases (3) and, therefore, may infer nsRP regardless of the severity of the trans allele (as seen here).

### 3.5. Patient 5 (GC22929)

Patient 5 is a 55-year-old Black Caribbean female with a lifelong history of hearing loss and RP consistent with a clinical diagnosis of USH2. There is no known family history of vision or hearing problems.

WGS and the subsequent clinical analysis pipeline analysis revealed a heterozygous previously reported pathogenic variant in *USH2A*c.2299del: p.(Glu767SerfsTer21), (ClinVar ID: 2351). An additional *USH2A* analysis identified a single candidate non-coding variant, c.9055 + 389G>A, with a SpliceAI (Δ) score of 0.29 for a donor site gain (GTAGG>GTAAG) with an additional acceptor activation 80bp upstream, suggesting a possible 78bp pseudoexon inclusion similar to findings in Patients 1 and 4. Consequently, this variant was selected for further functional investigation.

**Figure 5:**
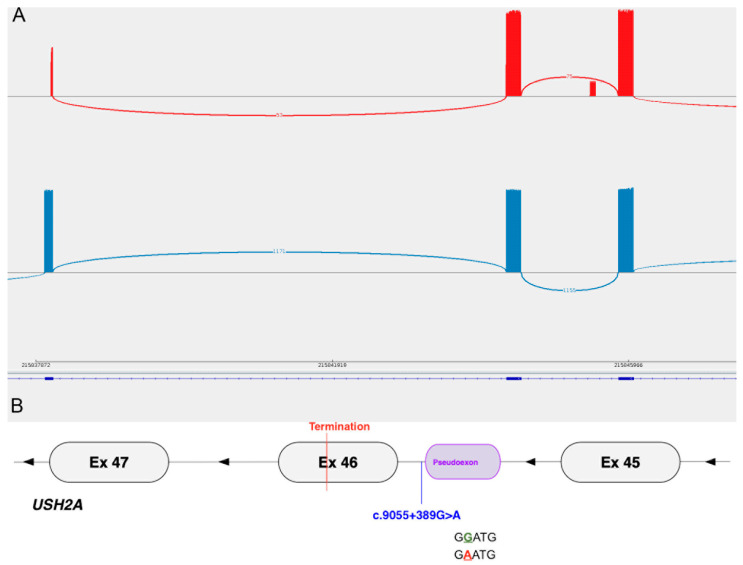
(**A**) Sashimi plot showing the splice junctions of Patient 5 (red, top) and a control (bottom, blue). *USH2A* exons 45–47 are depicted from right to left (reverse strand, 3′ to 5′) with each bar representing an exon and the height of the bars indicating the number of reads. The control sample (bottom, blue) shows only canonical splicing. The patient (top, red) shows mis-splicing—a pseudoexon inclusion of 78 bp in intron 45. The blue line at the bottom represents the reference *USH2A* transcript (NM_206933.4). The overall read depth was low due to short flow cell run time and low template in the library preparation. (**B**) Graphical representation of the mis-splicing pattern in Patient 5 showing the mutant donor site motif.

The RT-PCR and ONT sequencing of a 702 bp transcript region spanning exons 43–47 in Patient 5 revealed a 78 bp pseudoexon inclusion in 17.5% (17/97) of the reads, corresponding to the predicted outcome (Figure 5). This pseudoexon inclusion is predicted to lead to the premature termination of 65 codons downstream in exon 46, likely leading to the LOF of the protein (Table 1) in keeping with a clinical diagnosis of USH2 and seen in trans with a severe allele, p.(Glu767SerfsTer21). However, this does not take into account the low level of mis-spliced transcripts, similar to Patient 2. This may be attributed to the preferential allele amplification or NMD of the resultant transcript.

## 4. Discussion

This study provides insights into the genetic complexities of *USH2A* pathogenic genotypes in five patients who were initially molecularly unresolved. Following the re-analysis of WGS data, three patients were found to harbour deep intronic cryptic splice variants. Another had a synonymous variant previously categorised as a VUS, and one exhibited a seemingly biallelic null genotype without the expected hearing loss. The functional investigation using RT-PCR and subsequent nanopore sequencing analysis of NEC-derived *USH2A* transcripts confirmed the predicted impact on splicing, showing high concordance with the SpliceAI predictions, thus supporting the upgrade of these variants from VUS to likely pathogenic or pathogenic and providing a confirmed molecular diagnosis for the affected families. We demonstrate the clinical utility of ONT long-read sequencing for the characterisation of splice defects from specimens collected during routine clinic visits—with its single-molecule, long-read sequencing approach, this rapid and efficient method provides insights into complex splicing defects that would be difficult to investigate using traditional methods, such as short-read or Sanger sequencing. It further allows for the phasing of transcripts (e.g., Patient 2), which may be important for assessing incomplete splice defects.

According to the *USH2A* model of allelic hierarchy proposed by Lenassi et al., patients with two ‘null’ alleles (variants in trans that lead to protein truncation and/or NMD) present with USH2 due to the loss of functional protein, whereas patients with at least one ‘retinal disease-specific’ allele (missense/splice site-altering/other variants not leading to significant protein truncation) undergo normal cochlear development due to the presence of a partially functional protein [4]. Accordingly, given Patient 2’s clinical diagnosis of USH2, the anticipated genotype would include two LOF variants, and indeed, the trans pathogenic variant, c.7595-2144A>G, identified in this patient, is a well-characterised deep intronic variant that is predicted to result in LOF [22]. However, the candidate synonymous variant c.2139C>T identified in this patient was shown in our experimental studies to result in the partial skipping of exon 12 in 26% of the reads. As such, the dominant in-frame effect seen on the transcript analysis is milder than expected. It is possible that the relatively low proportion of altered transcripts vs. wild-type sequencing reads (confirmed to be from the trans allele) could be attributed to NMD-reducing transcript levels from the mutant allele, potentially due to a frameshift effect observed in a small proportion of reads. Patient 5 similarly displayed low levels of the mutant allele (18%); in this case, the predominant effect of the variant is predicted to lead to LOF, which could explain the significantly low proportion of the mis-spliced transcript.

Patient 3 harboured two *USH2A* variants, both predicted to cause LOF—c.4222C>T; p.(Gln1408Ter) and c.3812-3_3837dup; p.(Met1280Ter)—and yet had a phenotype inconsistent with her genotype (isolated RP with no hearing loss at an advanced age). The splice analysis of the exon 17–20 region revealed multiple mis-splicing events—our data demonstrated that the major detectable effect was the in-frame skipping of exon 18. In contrast to that seen in Patients 2 and 5, it may be that transcripts resulting in the exon 18 stopgain are subject to NMD, reducing their level of detection (seen at only 1.8% read depth); however, it could also be that the dominant presence of the in-frame transcript is responsible for driving the milder clinical presentation in Patient 3. These findings illustrate that variants impacting coding regions can also influence splicing in ways that are not always immediately evident (18) and can lead to unexpected genotype–phenotype associations. Cases 2, 3, 4, and 5 illustrate the difficulty in assessing the level of effect on the transcript with between 18% (patient 5) and 100% (patient 4) of the reads showing an effect on splicing. The apparent allele imbalance seen may have several contributing factors, including inherent preferential reverse transcription or amplification due to random hexamer use, secondary structures, primer binding efficiency, or the viability of altered transcripts and NMD of transcripts derived from either allele. It is, therefore, clear that the method has limitations in its quantification of effects.

A major challenge in investigating and interpreting putative disease-causing non-coding variants in the clinical setting centres around the difficulties in the functional validation of splice outcomes. Many retinal dystrophy genes show tissue-specific expression exclusively in photoreceptors, with the limited accessibility of retinal tissue for biopsy. Mini- or midi-gene expression vectors are often used to overcome this limitation; however, these artificial constructs only represent a small fragment of the sequence and may not accurately replicate the true splice environment of the gene in vivo. Additionally, creating and testing these vectors is an expensive and time-consuming process [23]. As a result, many variants remain of uncertain significance, leaving patients without a confirmed molecular diagnosis.

We have demonstrated that the RT-PCR and nanopore sequencing of NEC-derived mRNA is a reliable, minimally invasive, and effective approach for the study of putative splice-altering variants in *USH2A* transcripts. ONT long-read sequencing allows for the transcript phasing of heterozygous coding single-nucleotide variants and provides an assay to detect multiple mis-splicing events where present, all in a single experiment. This provides a more detailed view of the splicing landscape consequent upon different examples of cryptic splice variants for the most common cause of both USH2 and arRP in a large tertiary inherited retinal disease service, highlighting its suitability for use within routine clinical diagnostic pipelines for splice analysis. There may be occasions, however, where recalling patients for additional sampling may not be feasible, and there is, therefore, a significant clinical need in the era of routine whole genome sequencing for clinical diagnostics for effective and clinically validated in silico tools that can empower clinical laboratories to confidently classify such variants. Our study demonstrates the efficacy of SpliceAI in predicting the outcome of putative pathogenic alleles, highlighting its utility in accurately predicting complete splice outcomes, including the accurate prediction of pseudoexons. For the duplication, the major splicing outcome observed was exon skipping with low-level alternate splice acceptor usage; this was in keeping with the SpliceAI prediction (∆ 0.57 acceptor loss vs. ∆ 0.29 acceptor gain).

Cryptic splice-altering deep intronic variants are likely underestimated due to a lack of established evidence for pathogenicity, potentially representing a significant mechanism of disease in patients who remain molecularly undiagnosed following coding region analysis. The identification and functional validation of deep intronic variants in *USH2A* will provide affected individuals with a confirmed molecular report; these variants are important to identify given that they may be amenable to emerging antisense-oligonucleotide therapies that can potentially restore the normal function of the protein [12,22]. It should be noted, however, that for a defined gene/disease association such as *USH2A*:USH2/arRP, and with the careful selection of patients for the study, the diagnostic yield in our study is understandably higher. It is likely much more difficult to prioritise non-coding variants in patients where there are no clinical or genetic clues pointing to a culprit gene.

In conclusion, we provide evidence for pathogenicity for four VUSs identified in *USH2A* following an inconclusive genetic investigation. In addition, we demonstrate the in-frame splice alteration for a variant previously reported to be LOF in a patient with nsRP. We demonstrate the clinical utility of ONT long-read sequencing for characterising splice defects via RT-PCR and the sequencing of amplicons. This rapid, efficient method offers valuable insights into complex splicing defects that would be challenging to investigate via traditional methods and allows for simultaneous variant phasing in the presence of a coding SNV, including synonymous variants with a predicted effect on splicing.

## Figures and Tables

**Table 1:** Summary of variants selected for study.

Patient (ID)	Candidate Variant (GRCh38, NM_206933.4)	Previously Reported	Gnomad Frequency (v4.1.0)	ClinVar Prediction	Predicted Protein Consequence	Observed Protein Consequence
1 (GC20114)	chr1:g.215793253G>A c.9959-2971C>T	No	Absent	Absent	p.(Met3321LeufsTer8)	p.Met3321LeufsTer8
2 (GC19437)	chr1:g.216250931G>A c.2139C>T	Yes [19]	0.00001549	Conflicting (VUS ×3, Pathogenic ×1)	p.(Gly713=)	p.Glu714_Gly723del
3 (GC2569)	chr1:g.216198560_216198588dup c.3812-3_3837dup	Yes (carrier state in an affected individual with an alternative molecular diagnosis) [20]	0.0007429	Conflicting (LP ×1, VUS ×2, LB ×1)	p.(Met1280Ter)	p.Gly1271_Ser1360del
4 (GC20993)	chr1:g.215867824G>C c.8682-654C>G	Yes [21]	0.000006570	Absent	p.(Phe2895Ter)	p.Phe2895Ter
5 (GC22929)	chr1:g.215845435C>T c.9055+389G>A	No	0.00001971	Absent	p.(Glu3019AspfsTer65)	p.Glu3019AspfsTer65

VUS: variant of uncertain significance, LP: likely pathogenic, LB: likely benign.

**Table 2:** *USH2A* genotype summaries for all patients.

Patient (ID)	Clinical Diagnosis	*USH2A* Variants (NM_206933.4)	
		Variant 1	Variant 2
1 (GC20114)	USH2	c.9959-2971C>T	c.6289_6302del14 p.(Ile2097Ter)
2 (GC19437)	USH2	c.2139C>T	c.7595-2144A>G
3 (GC2569)	RP	c.3812-3_3837dup	c.4222C>T p.(Gln1408Ter)
4 (GC20993)	RP	c.8682-654C>G	c.9882C>G p.(Cys3294Trp)
5 (GC22929)	USH2	c.9055 + 389G>A	c.2299del p.(Glu767SerfsTer21).

**Table 3:** SpliceAI predictions.

Patient and Variant	(Δ) Type	(Δ) Score	Position	Consequence
1 (GC20114) c.9959-2971C>T	Acceptor gain	0.68	+114	Pseudoexon inclusion
	Donor gain	0.67	+2	113 bp (intron 50)
2 (GC19437) c.2139C>T	Donor loss	0.89	−28	Partial exon skipping
	Donor gain	0.86	+2	30 bp (exon 12)
3 (GC2569) c.3812-3_3837dup	Acceptor loss	0.57	−3	Weakening of canonical site
	Acceptor gain	0.29	0	Use of first copy of the acceptor motif and stop gain within exon 18
4 (GC20993) c.8682-654C>G	Acceptor gain	0.36	+131	Pseudoexon inclusion
	Donor gain	0.50	+3	130 bp (intron 43)
5 (GC22929) c.9055+389G>A	Acceptor gain	0.26	+80	Pseudoexon inclusion
	Donor Gain	0.29	+4	78 bp (intron 45)

A (Δ) score of 0.20 or more was considered significant. The position indicates the location of the predicted splice effect relative to the variant, measured in base pairs (bp). (+) values occur downstream of the variant, and (−) values upstream of the variant location, consistent with the orientation of *USH2A* on the reverse strand.

## Data Availability

Research on the de-identified patient data used in this publication can be carried out in the Genomics England Research Environment subject to a collaborative agreement that adheres to patient-led governance. All interested readers will be able to access the data in the same manner that the authors accessed the data. For more information about accessing the data, interested readers may contact research-network@genomicsengland.co.uk or access the relevant information on the Genomics England website: https://www.genomicsengland.co.uk/research, accessed on 20 May 2024.
